# UK Breastfeeding Helpline support: An investigation of influences upon satisfaction

**DOI:** 10.1186/1471-2393-12-150

**Published:** 2012-12-13

**Authors:** Gill Thomson, Nicola Crossland, Fiona Dykes, Chris J Sutton

**Affiliations:** 1 Maternal and Infant Nutrition and Nurture Unit (MAINN), School of Health, University of Central Lancashire, Preston, PR1 2HE, England; 2 School of Health, University of Central Lancashire, Preston, PR1 2HE, England

**Keywords:** Breastfeeding, Helpline, Survey, Descriptive, Evaluation, Peer support

## Abstract

**Background:**

Helpline services have become an increasingly popular mode of providing community access to information and expert information and advice in the health and welfare sector. This paper reports on data collected from 908 callers to UK-based breastfeeding helplines.

**Methods:**

A mixed methods design was adopted utilising a structured interview schedule to elicit callers experiences of the help and support received. In this paper we report on a series of multiple regression models undertaken to elicit the variables associated with callers’ ‘overall satisfaction’ with the helpline service. Three models were constructed; 1) caller demographic/call characteristics; 2) attitudes and effectiveness of service characteristics and 3) impact of support on caller wellbeing.

**Results:**

Overall, 74.6% of callers were very satisfied, and 19.8% were satisfied with the help and support received by the helpline service. The caller demographic/call characteristics found to have a significant relationship with overall satisfaction related to the ease of getting through to the helpline and whether the woman had previously breastfed. Service characteristics associated with overall satisfaction related to whether the information received was helpful and whether the support helped to resolve their issues. The extent to which the volunteer was perceived to have enough time, whether the support had encouraged them to continue breastfeeding, met the caller’s expectations and/or provided the support the caller needed were also significantly associated. Caller outcomes contributing significantly to overall satisfaction concerned callers feeling less stressed, more confident, reassured and determined to continue breastfeeding following the call. Consideration of the effect sizes indicated that key factors associated with overall satisfaction related to: volunteers having sufficient time to deal with the callers’ issues; the information being perceived as helpful; the volunteers providing the support the callers needed; and for callers to feel reassured following the call.

**Conclusion:**

Overall, these results highlight the value of the breastfeeding helpline(s) in terms of providing rapid, targeted, realistic, practical, and responsive support that provides affirmation and encouragement. The benefits include confidence building and callers feeling reassured and motivated to continue breastfeeding. Care needs to be taken to ensure that helpline support is easily accessible to ensure that callers and their families can access support when needed. This may require consideration of extension to a 24 hour service.

## Background

Counselling and helpline services in the health and welfare sector have become an increasingly popular mode of providing community access to information and expert advice [1] and are considered to offer an important first point of contact for those seeking assistance [2]. Telephone support is perceived to be a low cost [3], flexible, private [4] and non-stigmatizing form of support [5]. This method of support is considered to reduce key barriers to health care, such as accessibility [6,7], particularly among those who may be less likely to utilise statutory services (i.e. younger service-users) [8]. Furthermore, access to telephone counselling may also empower individuals to seek help through more conventional treatment and support [4,6,9] as well as providing economic and resource benefits by reducing the number of consultations with medical/clinical professionals [1]. Currently there is a wide range of helpline services, across a number of countries, offering advice, information and support on a variety of public health issues such as problem gambling, smoking, alcoholism, and diabetes. Whilst some helplines offer specific medical advice (such as NHS Direct), others, mainly funded by the voluntary sector, tend to operate from an ethos of empowering callers to make their own decisions [10].

Helpline evaluations tend to be rare due to the anonymous nature of these services [2]. However existing published studies and reports commonly convey high levels of user satisfaction. An evaluation of the Parentplus helpline was undertaken by Boddy and colleagues [11]. The findings revealed that the vast majority of callers were very positive about the helpline service, with over 85% reporting that their situation had improved as a result of the call. Urbis Keys Young [12] evaluated three Australian helplines, Men’s Line, Care Ring and Lifeline to elicit what makes telephone counselling a satisfactory experience. Satisfaction was associated with whether service-users were provided with sufficient time; whether they were provided with good ideas, strategies and suggestions; accessibility of the support; and how respectful, skilful and professional the volunteers/counsellors were perceived to be [2]. A study by Lim et al. [13] found high levels of service-user satisfaction with the quality of information provided through the MotherSafe teratology information service in Australia. Dennis and colleagues [14] also assessed the impact of telephone-based peer support on reported levels of postpartum depression in Canada. The results identified that at 12 weeks postnatal, 15% in the intervention group compared to 25% in the control group had an Edinburgh postnatal depression scale score of >12 (a score of 12–13 identified as the optimum cut off for this scale to identify major depression). The results from this evaluation also identified that over 80% of mothers were very satisfied with the service, as well as reporting high levels of positive relationship qualities such as trust (83.6%) and perceived acceptance (79.1%) by the peer supporters [15]. Finally, a Cochrane review undertaken by Dale et al. [16] provided some evidence that peer support telephone calls can be effective for certain health-related concerns; however, generalisability of the findings was limited due to the methodological quality of the papers reviewed.

To date, projects have explored the utility of telephone-based breastfeeding support, with studies identifying that breastfeeding telephone support may influence breastfeeding rates [5,17]. A randomised controlled trial of telephone-based breastfeeding peer support for adolescent mothers was undertaken by Meglio et al. [18]; they identified that, whilst breastfeeding duration did not significantly differ between the intervention group and control group, the duration of exclusive breastfeeding was increased for those who received the intervention. Dennis et al. [19] conducted a randomized controlled trial to evaluate the effect of peer (mother-to-mother) telephone-based support on breastfeeding duration among first-time breastfeeding mothers. It showed that 81.6% of women were satisfied with the peer support service, and significantly more mothers in the intervention group than in the control group continued to breastfeed at three months post-partum (81.1% v. 66.9%, *p* = 0.01) and did so exclusively (56.8% v. 40.3%, *p* = 0.01). A recent study evaluated the UK-based Drugs in Breastmilk Helpline. This helpline provides information on the use of medications while breastfeeding and is used predominantly by mothers, but also by health professionals. The study reported high levels of user satisfaction with the service, with women commonly contacting the helpline seeking reassurance or information to resolve conflicting advice from health professionals [20]. A broad scope of the literature has suggested that there are no published reports into the efficacy of a generic breastfeeding helpline service. Studies such as those undertaken by Chamberlain et al. [21] and Wang et al. [22] report on the level of need and reasons for usage of a breastfeeding telephone support line; however, service-user evaluations of the telephone service were not included.

In the UK, a number of breastfeeding telephone helplines are in operation provided by four key organisations: the National Childbirth Trust (NCT), La Leche League (LLL), the Breastfeeding Network (BfN) and Association of Breastfeeding Mothers (ABM). All these helplines are operated by breastfeeding peer supporters, who are mothers who have/are currently breastfeeding, with calls directed to and taken in their own homes. All peer supporters must have completed an introductory and more advanced training course (e.g. up to 2 years) prior to taking calls on the helpline; followed up ongoing supervision and attendance at additional training sessions. Thousands of callers contact these helplines each year to receive timely practical information and support with breastfeeding.

Since February 2008, a National Breastfeeding Helpline (NBH) has been in operation, funded by the Department of Health. The helpline was established to provide an accessible, universal service available through a national number; providing supplementary support for breastfeeding women alongside services provided by the NHS, as well as peer support provision and/or breastfeeding groups in operation. This service offers opportunities for information, support (practical as well as emotional) and where appropriate signposting callers through to others sources of help and support. This service is currently provided by unpaid volunteers who are either a BfN Registered Breastfeeding Supporter, or an ABM Breastfeeding Counsellor.

The helpline service is available in England, Wales, Scotland and Northern Ireland, with lines open from 9.30am until 9.30pm every day. In the first instance, callers who call from a landline are routed through to their nearest trained volunteer; if this service is not available due to the volunteers/counsellors being busy with another caller and/or availability of local volunteers, the call is then routed through to a ‘default’ line. All callers who call from a mobile network are transferred direct to the ‘default’ line. Volunteers/counsellors who staff the ‘default’ line thereby respond to callers from anywhere across the UK.

The ABM and the BfN employ paid link workers (BfN) and regional coordinators (ABM) who coordinate the volunteers/counsellors who operate the helpline(s) from within specific geographical areas. There are a total of 10 link workers/regional coordinators (five employed by each organisation) who cover all the UK regional areas. As indicated above, the BfN and ABM both operate their own helpline service. When the NBH came into operation, rather than each organisation running two separate helpline services (i.e. their own and the NBH) a decision was made to integrate the helplines. In reality this means when a volunteer/counsellor is operating the helpline, they will receive calls from callers who have contacted the helpline of their own organisation (BfN or ABM respectively) or the NBH. The volunteer/counsellor is unaware as to which helpline number the caller has called, with all calls being dealt with in exactly the same manner.

Since the introduction of the NBH, the annual number of calls to its helpline has substantially increased (from approximately 9,000 in 2008 to over 35,000 in 2011), coupled with a marked decrease in the number of calls received by the BfN (from over 17,000 to approximately 12,000) and ABM (from over 7,000 to under 4,000) helplines over this time period Of the calls to NBH, approximately 35% (ranging from 34.2% in 2009 to 37.4% in 2011) are answered although it is important to note that up to 10% of calls are made outside of the opening hours. As the NBH had been running for approximately three years, the NBH commissioned our research unit to evaluate callers’ satisfaction and experiences with its service. In this paper we report on a series of multiple regression models undertaken to elicit the variables independently associated with callers ‘overall satisfaction’ of the helpline service. A further paper that addresses the qualitative insights and caller recommendations for service delivery is forthcoming.

## Methods

### Design

This project had a mixed-method design within a structured telephone interview to explore service-users’ satisfaction, experiences and perceptions of the breastfeeding helpline service. It was originally intended that this evaluation would solely focus on callers who had phoned the NBH service. However, due to the integration of the helplines, and the fact that callers receive a ‘usual’ service, a decision was made to recruit *any* callers who were using the helplines (ABM, BfN or NBH) and, where possible, try and elicit from the caller which helpline service they had contacted. Whilst health professionals use the helpline(s), as the study aimed to elicit the efficacy of the support and impact on caller outcomes, a decision was made to only recruit callers with direct experiences (e.g. mothers or a member of their personal network). We planned to sample at least 600 callers over a 14-week period using systematic random sampling, to undertake a minimum of 450 interviews. It was anticipated that, from approximately 4,500–5,000 calls received over a 14-week period, up to 50% would consent, providing a sampling frame of up to 2,500 from whom to select the sample of approximately 25%. It was decided to finalise the sampling strategy following a three-week pilot phase, during which all consenting callers would be included.

### Data collection tool design

A questionnaire-based structured interview schedule was used for the evaluation. Its primary purpose was to elicit callers’ attitudes towards issues such as: how easy it was to access the helpline (number of call attempts); how many times they had used the service; perceptions of the opening hours; reason for calling the helpline; attitudes towards and impact of the help and support received; follow-up support options provided; overall satisfaction and recommendations for service development. The questionnaire was designed and developed based on discussions with the funders, a review of the literature on helpline evaluations and the Health Technologies Assessment guidance on the design and use of questionnaires [23]. Drafts of the questionnaire were reviewed by the funders, six research-active members of the corresponding University, and trialled with four mothers. Subsequent revisions concerned question wording and sequence of questions rather than content changes.

The finalised questionnaire included a mixture of closed questions with a small number of options, many having five potential responses to attitudinal statements (sometimes with a ‘not applicable’ option to differentiate uncertainty from question irrelevance due to the nature of the call, e.g. breastfeeding discontinuation) and open questions. Basic demographic and personal details were also recorded including age (caller and child), marital status, ethnicity, parity as well as previous breastfeeding history. (Whilst it was acknowledged that the BfN and the ABM use different terms to denote their peer support provision, e.g. volunteer and counsellor respectively - volunteer was adopted as a generic term during this evaluation).

### Ethics

The proposal was reviewed and obtained ethical approval from the UCLan Faculty of Health Ethics Committee. Issues in relation to informed consent, confidentiality, anonymity and withdrawal were adhered to throughout the evaluation.

### Recruitment & sampling strategy

Over a 14-week period (w.c. 30.5.11 – w.c. 29.8.11) all the volunteers/counsellors operating the helpline were requested to ask callers (at the end of the call) whether they would be willing to take part in a telephone evaluation. Once agreement was obtained, the date and time of call, the caller’s name, telephone number, first half of their postcode, which helpline they had contacted (where known) and name of the provider organisation (ABM or BfN) was recorded. Each week, the details of these participants were collected from volunteers/counsellors by the link workers/regional coordinators, transferred to a master data form, and forwarded to the project lead (GT).

### Data collection

Duplicate names or callers’ details received more than three weeks after the call to the helpline were recorded and removed from the sample. A decision to remove the latter was to limit recall bias of callers’ attitudes and experiences. All remaining caller details were allocated to seven interviewers through distribution of a form that contained rows of caller data. Interviewers made up to four attempts to contact each of the callers, varying the time and day to increase the potential for making contact. Unless requested by the caller, each interview took place within three weeks of their call to the helpline.

At the start of the interview, a standardised script was used to provide introductions and information about the evaluation and to obtain verbal consent for the data collection. All questions within the structured interview schedule were then read out, with answers recorded on the interview form. At the end of the interview, the caller was asked whether they would like to receive a summary of the final evaluation report.

The project lead (GT) was notified of any incorrect telephone numbers and these were checked with the corresponding link workers/regional coordinators. Any occasions of where the participant was un-contactable or the caller was not willing to participate were recorded against the corresponding caller details on the data form.

All completed, anonymised interview schedules and updated/completed data forms were returned to the project lead on a weekly basis.

### Pilot phase

A three-week pilot was undertaken to provide an opportunity to reflect on the recruitment strategy, sampling plan and interview schedule. All pilot data were to be included in the final data set unless substantial changes to the interview schedule were made.

Data were collected from 75 callers over the three weeks and highlighted recruitment strategy concerns in terms of callers’ details not being received by all the link workers/regional coordinators. The funders were notified, with ongoing communication about this issue provided across the project. Minor alterations were made to the sequence and structuring of the questions to improve clarity and reduce ambiguity and repetition. However, as changes were minimal, it was decided to retain the pilot data in the full survey data set. Also, as response rates were lower and the duration of interviews was shorter than expected, we decided to include all consenting callers for the duration of the study.

### Reliability and validity of the data set

All interviewers were involved in a training session prior to data collection to ensure consistency in data recording. All the data was entered into SPSS v.19 and MAXQDA by the project lead (GT) on an ongoing basis. This enabled close monitoring of all completed scripts and ensured consistency in terms of how answers were being recorded (with ongoing feedback provided on an individual and group basis as appropriate). A 10% sample was checked for accuracy of coding (n=90) by a separate member of the evaluation team (NC), with the data errors totalling 0.73%.

### Data analysis

Analysis of quantitative data was performed using SPSS and Stata (v.11). The responses to the closed questions were analysed using descriptive analytic methods (frequencies and percentages for categorical data including Likert-type responses; median and inter-quartile range for age). Using linear modelling, a two-level hierarchical modelling approach was used to explore which factors independently affected ‘overall satisfaction’ scores. Due to low numbers in the ‘strongly disagree’ and ‘disagree’ categories for many of the variables, it was decided to combine these categories and, for consistency, to combine the ‘strongly agree’ and ‘agree’ categories. Caller demographic/call characteristics were considered at the first level of the hierarchy. Then two further models were fitted in which 1) attitudinal response to service characteristics and 2) outcomes of help and support on caller wellbeing were considered as additional explanatory factors at the second level. At each level, model selection was initially via a hierarchical backward elimination procedure of factors at that level, with all included terms at lower levels of the hierarchy included in all models considered at higher levels of the hierarchy. A 5% significance level was used for inclusion and exclusion of factors throughout; due to the large sample size it was decided that terms which were not significant at this level would not be important. For the model chosen at each level of the hierarchy, 95% confidence intervals were presented for the effects of each of the factors remaining in the model. Subsequently, alternative approaches to modelling (forward selection strategies for model selection and ordinal logistic regression) were applied to assess the sensitivity of findings to the analytic approach.

## Results

During June to August 2011 some 9,507 calls were made to the breastfeeding helpline(s), 3,529 of which were answered (37.1%). Over the 14-week evaluation period, 1,605 callers’ names were forwarded to the project lead. A total of 123 names were excluded due to either caller details having been received more than three weeks after their call to the helpline (n=99, 6.2%) or being duplicates (n=24, 1.5%).

Overall 1,482 names were forwarded across the interviewers. From this sample, a further 135 callers refused and/or were not willing or able to participate due to: incorrect telephone number recorded (n=78, 5.3%); caller not wanting to be interviewed (n=50, 3.4%); caller out of country/hospitalised (n=6, 0.4%); inappropriate call (student calling about coursework; n=1, 0.1%). A further 439 callers were not contacted due to either the caller not being available after four contact attempts or more than three weeks having elapsed since the index call.

A total of 908 telephone interviews were conducted. Thirteen interviews (1.4%) were only partially completed due to caller changing their mind, but all recorded data has been included in the analysis.

**Figure 1:**
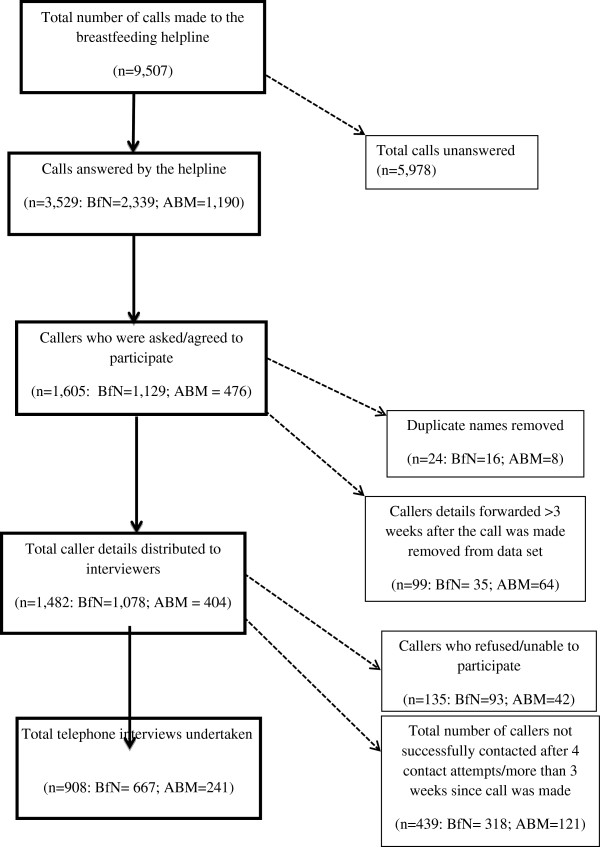
**Overview of recruitment**/**data collection process.**

A breakdown of total caller details and exclusions by provider (BfN and ABM) is presented in Figure 1.

Seven hundred and three (77.4%) callers reported having called the NBH, with 116 (12.8%) and 28 (3.1%) reporting having called the BfN and ABM helplines respectively and 61(6.7%) did not know which helpline they had called; this is relatively consistent with the overall call figures, with 73.8%, 21.8% and 4.4% of all helpline calls answered being to NBH, BfN and ABM, respectively, although this suggests that the NBH callers may have been over-represented and may have been more likely to recall which helpline they had called.

In the following sections, the descriptive data associated with a) caller demographic/call characteristics (model 1); b) attitudes and effectiveness of service characteristics (model 2); and c) callers outcomes (model 3) are reported. The results of the multiple regression models undertaken for the three identified models are then presented.

### Descriptive data

#### Caller demographic/call characteristic data

Overall, 885 telephone interviews were undertaken with mothers (97.5%), 17 with husbands/partners (1.9%), five with grandmothers (0.6%) and one with a sister (0.1%). The demographic data recorded concerned the age of the caller and details of the mother/child to whom the call was related was collected in terms of ethnicity, marital status, parity, age of child (to whom the call related) at the time of the call, current and previous (where appropriate) infant feeding practices and whether breastfeeding support had been provided by friends and family. Call characteristics data included which organisation dealt with the call, which helpline had been called, whether it was the first time they had called the helpline, the number of times the helpline had been accessed, the time of the call and callers’ perceptions of how easy or difficult it was to access the helpline. Descriptive statistics for all these variables are presented in Table 1:

**Table 1:** **Caller demographic**/**call characteristics descriptive statistics**: **frequency** (%) **unless otherwise stated**

**Caller demographic data**	**Frequency (%)**
**Age**	
Median (Interquartile range)	32 (29 – 35)
**Marital Status**	
Married/living together	858 (94.5%)
In relationship	17 (1.9%)
Single/separated/divorced	19 (2.1%)
Not recorded	14 (1.5%)
**Ethnicity**	
White	778 (85.7%)
Mixed	22 (2.4%)
Asian/Asian British	63 (6.9%)
Black/Black British	21 (2.3%)
Chinese/Other	9 (1.0%)
Not recorded	15 (1.7%)
**Parity**	
First-time mothers	607 (66.9%)
At least one previous child	288 (31.7%)
Not recorded	13 (1.4%)
**Age of child at time of call**	
Pregnant	5 (0.6%)
Under 1 month	446 (49.1%)
Between 1 – 5 months	340 (37.4%)
Between 6 – 12 months	86 (9.5%)
Over 12 months	16 (1.8%)
Not recorded	15 (1.6%)
**Current infant feeding practices**	
Exclusive/fully breastfeeding^1^	628 (69.2%)
Mixed feeding (breast and artificial milk)	189 (20.8%)
Formula feeding	68 (7.5%)
Not applicable^2^	10 (1.1%)
Missing	13 (1.4%)
**Did mother breastfeed previous child**/**children(n**=**301)**	
Breastfed previous child/children	241 (80.1%)
Did not breastfeed previous child/children	44 (14.6%)
Missing	16 (5.3%)
**Breastfeeding support from friends**/**family members**	
Received breastfeeding support	712 (78.4%)
Not received breastfeeding support	163 (18.0%)
Not sure/not relevant as pregnant	18 (2.0%)
Missing	15 (1.6%)
**Call Characteristics**	
**Which helpline called**	
NBH	703 (77.4%)
BfN	116 (12.8%)
ABM	28 (3.1%)
Don’t know	61 (6.7%)
**Provider organisation**	
BfN	667 (74.5%)
ABM	241 (26.5%)
**First-time caller**	
Yes	739 (81.4%)
No	166 (18.3%)
Missing	3 (0.3%)
**Number of times the helpline had been used**	
1–3 times	872 (96.0%)
4–6 times	21 (2.3%)
7–10 times	5 (0.6%)
10+ times	7 (0.8%)
Missing	3 (0.3%)
**When call was made**/**answered**	
9.30am up to 12 noon	313 (34.5%)
12 noon up to 6pm	349 (38.4%)
After 6pm	207 (22.8%)
Missing	39 (4.3%)
**How easy**/**difficult was it to get through to the helpline**	
Very easy/easy	800 (88.1%)
Neither easy not difficult	36 (4.0%)
Difficult/very difficult	72 (7.9%)

^1^ This includes 73 cases where the mother was breastfeeding and had introduced complementary foods.

^2^ Relates to mothers whose children were over 12 months of age and who had stopped breastfeeding, and mothers who were pregnant.

Callers into the helpline were typically aged between 29 and 35 years (with a median age of 32). Calls that concerned mothers who were married/living together (94.5%); of a white ethnic background (85.7%); who were first-time mothers (66.9%); whose infants were under one month of age (49.1%) and who were in receipt of breastfeeding support from friends or family (78.4%) were more common than other demographic categories. Calls that concerned multiparous mothers indicated that the vast majority (80.1%) had breastfed a previous child. Ninety percent of the mothers were providing their infants (either fully or partially) with breast milk at the time of the call to the helpline.

The call characteristics data indicates that the majority of calls were made into the NBH service, and were taken by volunteers from the BfN. Of the sample, 81.4% were first-time callers, 96% had accessed the service on between one and three occasions, with a fairly even distribution of calls across the day. Almost 90% of callers reported that it was easy or very easy to get through to the helpline service.

Details on the ‘reason for call’ were recorded during the interview. Over half of the callers (521; 57.4%) rang the helpline about a single issue; 269 (29.6%) reported two issues and just under 13% of callers had multiple (ranging from three to nine) issues. A total of 1,447 responses were recorded, categorised and are presented in Table 2.

**Table 2:** Reasons for calling the helpline service descriptive statistics

**Reason for call**	**Frequency (%)**
Difficulties with positioning and attachment	364 (40.1%)
Concerns about milk supply	274 (30.2%)
Difficulties relating to infant behaviour	268 (29.5%)
Medical issues	185 (20.4%)
Feeding other than by breastfeeding	147 (16.2%)
Social or emotional issues	81 (8.9%)
Other	128 (14.1%)

The most common category of reason for calling the helpline was ‘difficulties with positioning and attachment’, reported by 40.1% of callers. Overall 30.2% of callers contacted the helpline due to ‘concerns about milk supply’, including concerns about inadequate milk supply and/or poor infant weight gain, as well as oversupply and engorgement. Almost 30% of callers phoned the helpline about ‘difficulties relating to infant behaviour’ such as colic, crying, frequent feeding and reflux, and 20.4% of callers sought advice related to ‘medical issues’ such as mastitis, thrush, or questions about medication. ‘Feeding other than by breastfeeding’ (16.2%) included issues relating to expressing and storing breast milk, feeding with infant formula milk, bottle-feeding and complementary feeding. While almost all calls involved an element of emotional support, just under 9% of callers specifically contacted the helpline(s) for help with ‘social or emotional issues’ such as reassurance, resolution of conflicting advice, or advice about returning to paid work. ‘Other’ reasons (14.1%) comprises disparate issues such as stopping breastfeeding, re-lactating and the mother’s diet; this category also includes callers who either declined to disclose or could not remember their reason for calling the helpline.

#### Attitudes and effectiveness of service characteristics

During the interview, participants were asked to respond to a series of statements to ascertain the callers’ overall attitudes towards, and effectiveness of the help and support received in resolving their particular issue(s) (Table 3). Two feeder questions were also included in the schedule to determine whether a) participants were aware that all the volunteers were/had been breastfeeding mothers and b) whether they were aware that the volunteer was a local breastfeeding supporter. Those who answered in the affirmative to these statements were subsequently asked to report their satisfaction towards these features of the helpline support.

**Table 3:** **Perceptions of the service**: **attitudes**, **effectiveness and satisfaction**

**Statement**/**question**	**Frequency (%)**
**Were you aware that the volunteers were**/**had been breastfeeding mothers**?	
Yes	497 (54.7%)
No/not sure	411 (45.3%)
**Were you aware that the volunteer was a local breastfeeding volunteer**?	
Yes	242 (26.7%)
No/not sure	666 (73.3%)
**I liked being able to speak to another mum who had breastfed her own baby** (**n**=**497**)	
Strongly agree/agree	481 (96.8%)
Neither agree nor disagree	9 (1.8%)
Strongly disagree/disagree	5 (1.0%)
Missing	2 (0.4%)
**I liked being able to speak to a breastfeeding volunteer who knows the area where I live** (**n**=**242**)	
Strongly agree/agree	142 (58.7%)
Neither agree nor disagree	72 (29.8%)
Strongly disagree/disagree	26 (10.7%)
Missing	2 (0.8%)
**I felt the volunteer understood what I was talking about**	
Strongly agree/agree	867 (95.5%)
Neither agree nor disagree	14 (1.5%)
Strongly disagree/disagree	22 (2.4%)
Missing	5 (0.6%)
**I felt the volunteer understood what I**/**we were feeling**	
Strongly agree/agree	838 (92.3%)
Neither agree nor disagree	29 (3.2%)
Strongly disagree/disagree	24 (2.6%)
Not applicable	10 (1.1%)
Missing	7 (0.8%)
**I found it easy to talk about breastfeeding issues over the telephone**	
Strongly agree/agree	816 (89.9%)
Neither agree nor disagree	46 (5.0%)
Strongly disagree/disagree	40 (4.4%)
Not applicable	1 (0.1%)
Missing	5 (0.6%)
**I liked speaking to someone who didn**’**t know me**	
Strongly agree/agree	387 (42.6%)
Neither agree nor disagree	410 (45.1%)
Strongly disagree/disagree	105 (11.6%)
Missing	6 (0.7%)
**I felt comfortable talking about breastfeeding issues with the volunteer**	
Strongly agree/agree	895 (98.5%)
Neither agree nor disagree	2 (0.2%)
Strongly disagree/disagree	6 (0.7%)
Missing data	5 (0.6%)
**I felt that the volunteer had enough time for me**	
Strongly agree/agree	885 (97.4%)
Neither agree nor disagree	8 (0.9%)
Strongly disagree/disagree	10 (1.1%)
Missing	5 (0.6%)
**I felt that the volunteer treated me with respect**	
Strongly agree/agree	895 (98.6%)
Neither agree nor disagree	2 (0.2%)
Strongly disagree/disagree	4 (0.4%)
Missing	7 (0.8%)
**I felt that the volunteer gave me**/**us the support that I/we needed**	
Strongly agree/agree	844 (93.0%)
Neither agree nor disagree	23 (2.5%)
Strongly disagree/disagree	35 (3.8%)
Missing	6 (0.7%)
**The support received met my expectations**	
Strongly agree/agree	825 (90.9%)
Neither agree nor disagree	28 (3.1%)
Strongly disagree/disagree	42 (4.6%)
Not applicable	4 (0.4%)
Missing	9 (1.0%)
**The volunteer made me**/**us feel it was OK to carry on breastfeeding**	
Strongly agree/agree	846 (93.1%)
Neither agree nor disagree	6 (0.7%)
Strongly disagree/disagree	7 (0.8%)
Not applicable	42 (4.6%)
Missing	7 (0.8%)
**The volunteer encouraged me**/**us to continue breastfeeding**	
Strongly agree/agree	776 (85.5%)
Neither agree nor disagree	29 (3.2%)
Strongly disagree/disagree	12 (1.3%)
Not applicable	83 (9.1%)
Missing	8 (0.9%)
**I felt that the volunteer was an** ‘**expert**’ **in breastfeeding issues**	
Strongly agree/agree	796 (87.7%)
Neither agree nor disagree	74 (8.1%)
Strongly disagree/disagree	31 (3.4%)
Missing	7 (0.8%)
**The information I received from the volunteer was helpful**	
Strongly agree/agree	855 (94.1%)
Neither agree nor disagree	19 (2.1%)
Strongly disagree/disagree	28 (3.1%)
Missing	6 (0.7%)
**I felt that the volunteer was able to answer my questions**	
Strongly agree/agree	838 (92.3%)
Neither agree nor disagree	36 (3.9%)
Strongly disagree/disagree	29 (3.2%)
Missing	5 (0.6%)
**I**/**we were able to put into practice the information provided**	
Strongly agree/agree	778 (85.7%)
Neither agree nor disagree	31 (3.4%)
Strongly disagree/disagree	50 (5.5%)
Not applicable	41 (4.5%)
Missing	8 (0.9%)
**The support helped to resolve the issues**	
Strongly agree/agree	681 (75.0%)
Neither agree nor disagree	71 (7.8%)
Strongly disagree/disagree	128 (14.1%)
Not applicable	20 (2.2%)
Missing	8 (0.9%)
**The support I**/**we received encouraged us to continue breastfeeding**	
Strongly agree/agree	771 (84.9%)
Neither agree nor disagree	21 (2.3%)
Strongly disagree/disagree	31 (3.4%)
Not applicable	77 (8.5%)
Missing	8 (0.9%)
**I**/**we would not have been able to carry on breastfeeding if the helpline had not been contacted**	
Strongly agree/agree	188 (20.7%)
Neither agree nor disagree	78 (8.6%)
Strongly disagree/disagree	528 (58.2%)
Not applicable	106 (11.6%)
Missing	8 (0.9%)

Overall these data reflect high rates of satisfaction towards the helpline service. Some 96.8% of the callers (who were aware of this feature of the helpline service) liked having the opportunity to discuss breastfeeding with someone who had themselves breastfed. Furthermore, 95.5% and 92.3% of callers strongly agreed/agreed that the volunteer understood what they were talking about and how they were feeling respectively. Some 89.9% of callers found it easy to talk about their breastfeeding issues over the telephone; just over 40% liked the anonymous nature of the service and 98.5% of callers felt comfortable talking about their breastfeeding issues with the volunteer. Almost all of the callers (97.4%) considered that the volunteer had enough time for them and that the volunteer treated them with respect (98.6%).

Ninety-three percent of the callers felt that the helpline service had provided them with the support they needed, with just over 90% of callers considering the support to have met their expectations. The results indicated that 87.7% of participants considered the volunteer to be an ‘expert’ in breastfeeding issues and 92.3% of callers strongly agreed/agreed that their question(s) had been answered. Moreover, almost all of the participants (94.1%) agreed that the information they received had been helpful; 85.7% felt that they had been able to put into practice the information provided; with 75% considering that the support had helped them resolve their issues. Whilst only approximately 20% considered the helpline to have made a significant impact on breastfeeding continuation, it is important to note that approximately 85% considered that the volunteer and/or support they received had encouraged them to continue breastfeeding and 93.1% considered that the volunteer had made them feel it was OK to continue breastfeeding.

#### Outcome variables (overall satisfaction, caller wellbeing and follow-up support options provided)

Callers were asked to report their overall satisfaction of the helpline service, whether they would use the service again, and recommend to others. A series of statements were also posed to elicit whether the support received via the helpline had had any personal benefits in terms of improving callers’ wellbeing, their knowledge base and/or motivation to continue breastfeeding following the call. Further questions also explored whether the caller/mother had been provided with follow-up support options in terms of re-contacting the helpline service or accessing local support options (such as through health professionals, web sites or breastfeeding support groups). Details of the statements and question posed together with a summary of the responses are detailed in Table 4.

**Table 4:** **Overall satisfaction**, **caller wellbeing and follow**-**up support**

**Overall Satisfaction**	**Frequency (%)**
**How would you rate your overall satisfaction with the breastfeeding helpline**	
Very satisfied	677 (74.6%)
Satisfied	180 (19.8%)
Neither satisfied not dissatisfied	18 (2.0%)
Dissatisfied	16 (1.7%)
Very dissatisfied	6 (0.7%)
Missing	11 (1.2%)
**Would you use the helpline again**?	
Yes	855 (94.2%)
No	31 (3.4%)
Not sure	11 (1.2%)
Missing data	11 (1.2%)
**Would you recommend the helpline to others**?	
Yes	872 (96.0%)
No	19 (2.1%)
Not sure	5 (0.6%)
Missing data	12 (1.3%)
**Caller wellbeing**	**Frequency (%)**
**Following the call did you feel**	
**Less worried**	
Strongly agree/agree	801 (88.2%)
Neither agree nor disagree	25 (2.7%)
Strongly disagree/disagree	54 (6.0%)
Not applicable	19 (2.1%)
Missing	9 (1.0%)
**Less stressed**	
Strongly agree/agree	783 (86.2%)
Neither agree nor disagree	27 (3.0%)
Strongly disagree/disagree	52 (5.7%)
Not applicable	37 (4.1%)
Missing	9 (1.0%)
**More confident**	
Strongly agree/agree	772 (85.0%)
Neither agree nor disagree	46 (5.1%)
Strongly disagree/disagree	56 (6.2%)
Not applicable	25 (2.7%)
Missing	9 (1.0%)
**Reassured**	
Strongly agree/agree	835 (92.0%)
Neither agree nor disagree	19 (2.1%)
Strongly disagree/disagree	39 (4.3%)
Not applicable	5 (0.5%)
Missing	10 (1.1%)
**More knowledgeable about breastfeeding**	
Strongly agree/agree	701 (77.2%)
Neither agree nor disagree	56 (6.1%)
Strongly disagree/disagree	103 (11.4%)
Not applicable	38 (4.2%)
Missing	10 (1.1%)
**More determined to continue breastfeeding**	
Strongly agree/agree	631 (69.5%)
Neither agree nor disagree	69 (7.6%)
Strongly disagree/disagree	78 (8.6%)
Not applicable	118 (13.0%)
Missing	12 (1.3%)
**Follow**-**up support offers**	
**I**/**we were encouraged to call the helpline again** (**if issues persist or new issues present**)	
Yes	750 (82.6%)
No	71 (7.8%)
Not sure / can’t remember	74 (8.2%)
Missing	13 (1.4%)
**Additional support options provided**	
Yes	487 (53.6%)
No – not needed	133 (14.7%)
No/not sure	277 (30.5%)
Missing	11 (1.2%)

These data reveal that almost 95% of callers were satisfied/very satisfied with the helpline service; 94% identified that they would use the service again in the future and 96% would recommend this service to others. The helpline made a positive impact on caller wellbeing, with callers feeling less worried (88.2%), less stressed (86.2%) and more confident (85%) following the call. Almost all of the participants (92%) claimed that they felt reassured, and some 77.2% of the participants considered themselves to be ‘more knowledgeable’ about breastfeeding after their call to the helpline. Furthermore, 69.5% reported that they were more determined to continue breastfeeding after the call had been made. With regard to follow-up support options, over 80% of callers reported having been encouraged to call the helpline again, and approximately 53% of callers reported that they were signposted into additional support options, with a further 14.7% reporting that additional follow up had not been needed due to the support they had received via the helpline.

### Multiple regression models

A series of multiple regression models were undertaken to investigate which, and how, variables affected the callers’ ‘overall satisfaction’ of the helpline service.

#### Model 1: caller demographics/call characteristics

The first model explored the impact of caller demographics and call characteristics on overall satisfaction. These were investigated first, as these were personal characteristics and choices made by the caller and were not influenced by the experiences during the call. Caller characteristics included were age of caller, ethnicity (data re-coded as ‘white’ and non-white), age of infant at time of call, whether mothers had received breastfeeding support from friends and/or family members, parity, whether the infant was receiving any breast milk at the time of the evaluation and whether mother had breastfed before (the data was re-coded into two groups, a) primiparous mothers and mothers who had not breastfed previously and b) mothers who had breastfed previously (as initial exploratory modelling identified similar effect sizes between these groups). Marital status was not included in the analysis due to the vast majority of respondents being married/living together/in relationship. Call characteristics included which helpline was called, which organisation answered the call, whether it was the first time the caller had used the helpline, the time the call was answered, how many times the helpline had been used and callers’ attitudes towards how easy/difficult it was for their call to be answered.

Terms eliminated from the model in turn were parity (p=0.91), ethnicity (p=0.78), provider (p=0.73), infant provided with breast milk at time of call (p=0.69), how many times the helpline had been utilised (p=0.57), breastfeeding support provided by friends/family (p=0.53), first time caller (p=0.46), caller’s age (p=0.56), child’s age (p=0.38), helpline called (p=0.26) and time of call (p=0.18). The resulting model (Additional file 1: Table S1) suggested that only two factors: 1) whether the mother had breastfed previously (p=0.005) and 2) callers attitudes in regard to how easy/difficult it was to access the helpline support (p<0.0001) explained the differences between callers’ overall satisfaction. Mothers who had breastfed previously had, on average, 0.14 (95% CI 0.04 to 0.24) higher satisfaction scores than those who had not (as first-time mothers or having formula fed previous children). Those finding the breastfeeding helpline easy to access were significantly more satisfied than those who found it difficult to access, scoring an average 0.25 (95% CI 0.08 to 0.41) higher satisfaction score. However, those who were ambivalent in their attitude towards ease of access to the helpline scored 0.54 (95% CI 0.32 to 0.76) better than those who found the helpline difficult to access. This result could be explained by the fact that callers often reflected on all their experiences of accessing the helpline during the evaluation, whilst their attitudes towards the help and support they received via the helpline were focused on their recent call (the call when they were recruited to participate in the study). First-time callers were more likely than repeat callers to express uncertainty about the ease of accessing the helpline (9.0% vs 3.0%, p=0.001) therefore may represent those who had had varied experiences (positive and negative) in contacting the helpline, hence the callers were more dissatisfied overall towards the service. Only 3.9% of the variability in overall satisfaction was explained by this model, suggesting that caller characteristics played only a small part in determining overall satisfaction. This is, of course, reassuring as one would hope and expect that satisfaction is determined by the callers’ experiences of the service during the call, rather than any underlying characteristics of the caller or the corresponding call. Alternative model fitting strategies led to selection of the same two explanatory factors.

#### Model 2: attitudes and effectiveness of service characteristics

The regression model was then expanded to explore how callers’ experiences (attitudinal responses to the help and support they received) affected their overall satisfaction, given the callers’ characteristics identified as important (i.e. previous breastfeeding experience and easy of getting through to the helpline).

Initially, the two-factor demographic/call characteristic model were retained within the analysis, with the various attitudinal variables (as listed in Table 3, with the exception of the questions related to the volunteer being local and having been a breastfeeding mother, due to the low awareness of these aspects of the service) individually included to determine their relationship with satisfaction. Following a backward elimination process, the factors removed in turn were: the ‘expert’ status of the volunteer (p=1.0); the volunteer being able to answer all the caller’s questions (p=0.63); the volunteer understanding how they were feeling (p=0.51); finding it easy to talk to the volunteer (p=0.42), whether the volunteer encouraged the caller/mother to continue breastfeeding (p=0.43); speaking to someone who does not know them (p=0.37); whether they felt comfortable discussing the issue with the volunteer (p=0.20), whether they felt the volunteer respected them (p=0.18); whether they would been unable to carry on breastfeeding otherwise (p=0.11); whether they felt the volunteer understood what they were talking about (p=0.12); whether the support provided encouraged them to continue breastfeeding (p=0.10); whether they were able to put into practice the advice received (p=0.054). The other six factors, together with the two factors from the first level of the hierarchy, remained statistically significant and this eight-factor model explained 64.7% of the variability in the overall satisfaction (Additional file 1: Table S1).

The attitudinal variables associated with overall satisfaction related to the volunteer having enough time (p<0.0001); the information received was helpful (p<0.0001); the volunteer providing the support the caller needed (p<0.0001); whether the support met the caller’s expectations (p<0.0001); whether the volunteer made the caller feel OK to carry on breastfeeding (p=0.001) and whether the support helped to resolve the caller’s issue(s) (p<0.0001). Consideration of the effect sizes suggests that key areas of satisfaction related to the time the callers were provided with, how helpful callers found the information and the extent to which the volunteer provided the support the callers’ needed, with these callers’ respectively scoring 1.06 (95% CI 0.77 to 1.34), 0.76 (95% CI 0.54 to 0.98) and 0.94 (95% CI 0.76 to 1.13) higher than those who disagreed with these statements. These findings thereby emphasise the importance of targeted support on satisfaction rates.

There was some sensitivity to the choice of modelling approach. Whilst all identified factors included in the model using a backward elimination approach were chosen, when forwards selection was used, the ‘expert’ status of the volunteer (p=0.032) and whether they felt the volunteer respected them (p=0.046) were both included. For the ‘expert’ status, effect sizes were 0.19 (95% CI −0.01 to 0.38) for strongly agree/agree – disagree/strongly disagree and 0.13 (95% CI 0.01 to 0.25) for strongly agree/agree – neither agree nor disagree; for volunteer respect, they were 0.34 (−0.11 to 0.78) for strongly agree/agree – disagree/strongly disagree and −0.61 (−1.21 to 0.00) for strongly agree/agree – neither agree nor disagree. There was minimal impact on the effects of the model’s other factors and the AR^2^ increased to 65.0%. Using ordinal logistic regression, the same model terms were included using backward elimination as when this approach was used with linear regression; however, whilst the ‘expert’ status of the volunteer entered forward selection, the volunteer’s apparent respect for the caller did not.

#### Model 3: caller wellbeing

A further model was fitted to determine whether overall satisfaction was influenced by key outcome variables in terms of the caller’s wellbeing; namely callers feeling less worried, less stressed, more confident, reassured, more knowledgeable and more determined to breastfeed.

A similar process was undertaken in which the two significant factors from the model 1 were retained and all caller wellbeing variables were considered using a backward elimination approach. Variables removed, in turn, were: the extent to which callers considered themselves more knowledgeable (p=0.26); and the callers feeling less worried (p=0.14). The selection of terms for the model was not sensitive to model selection process (backward elimination or forward selection) or type of model (linear or ordinal).

Whilst not considered for removal from the model, it is worth noting that the mother having breastfed before did not remain significant (p=0.13). The loss of significance of this variable in this extended model means that it is not an independent explanatory factor for satisfaction when these individual outcome factors are added to the original two-factor model. This suggests that some or all of the service characteristic attitudinal factors provide the explanation as to why those who had breastfed previously expressed greater overall satisfaction than those who had not breastfed previously.

All other terms remained highly significant (p<0.001). This indicated that callers feeling less stressed, more confident, more reassured and more determined to continue breastfeeding were the key wellbeing factors which impacted on overall satisfaction. Furthermore, consideration of the effect sizes revealed that reassurance was a key factor associated with overall satisfaction, with callers scoring 0.91 (95% CI 0.67 to 1.16) higher than those who disagreed with this statement.

## Discussion

This evaluation identified high overall satisfaction, and high rates of satisfaction towards almost all features of the helpline service. Statistical modelling also enabled clarification of which types of support, and the implications of this support on caller wellbeing, are most strongly linked to overall satisfaction rates. The multiple regression models identified that the mothers who had previous breastfeeding experiences were more satisfied with the service than mothers with no previous breastfeeding experiences. Those who found it easier to access the helpline were also more satisfied than those who rated it as difficult and, in particular, than those who had a mixed/ambivalent rating of access to this support. In relation to service characteristics, the actual utility of the support (via the time afforded, the information perceived as helpful, the support being delivered in such a way that it encouraged callers/women to continue breastfeeding, whether the support met the callers’ expectations and the transferability and usefulness of support on resolving breastfeeding concerns and issues) were significantly associated with overall satisfaction. Differences between callers’ overall satisfaction were also explained by callers’ reports of wellbeing in terms of reassurance, stress, confidence and determination to continue breastfeeding. Key attitudinal and outcome variables associated with overall satisfaction concerned the time the volunteers provided to the callers, how helpful the information was, callers being provided with the support that they needed, and for callers feeling reassured following the call.

### Study limitations

Whilst a substantial number of callers were involved in the evaluation study, one limitation of the study is the potential for sampling bias. The data indicates that only 45% of callers who contacted the helpline over the recruitment period agreed to take part. A number of the callers not recruited may have been health professionals (who were excluded from the study), repeat callers, or they had refused to take part. However, it may be that callers who were very distressed during the call and/or the call was not particularly positive may not have been recruited, thereby restricting participation to those who the volunteers/counsellors considered to be ‘satisfied’ with the call. A further possible limitation may concern how the interview was conducted and recorded across the various members of the evaluation team. Whilst a training session was organised, all recording and coding of the data was undertaken by the project lead, and feedback provided to all interviewers on an ongoing basis to ensure consistency, this does not eradicate the potential bias of how questions were presented, and the extent to which response options were emphasised or repeated during the interview. The multiple linear regression modelling was made under the assumption that the overall satisfaction was assessed using an interval scale (meaning that the ‘gaps’ or ‘intervals’ between points on the scale are of the same size). As the scale was designed to have a middle category (neither satisfied nor dissatisfied) we believed that this assumption was reasonable to make, supported by the similarity of the set of model terms selected when this assumption was relaxed when the ordinal logistic regression sensitivity analysis was performed. Our first level model and caller wellbeing model (model 3) were also insensitive to the approach used to select model terms, although the model using the attitudes and effectiveness of service characteristics (model 2) showed some sensitivity to model terms when different modelling and selection approaches were used, possibly due to the larger number of inter-related terms considered. However, it would appear that the caller’s perception of the volunteers’ expert status was important, with its inter-relationship with other factors causing its elimination early in the process, with it being important in explaining satisfaction only being detectable once other less relevant terms were removed. The conflicting findings from alternative modelling approaches mean that it is less clear whether the caller’s level of belief that the volunteer respected them was actually important; this would merit investigation in future studies.

### Attitudinal and outcome variables: association with overall satisfaction

With regard to attitudinal and outcome variables not associated with overall satisfaction, these findings would suggest that the volunteers’ knowledge, their ability to forge relationships, demonstrate respect and empathise with the callers and the anonymous nature of the service were not key facets of satisfaction. These results are dissimilar to previous evaluation studies which identified the importance of respect, trust and acceptance on satisfaction rates [12,14]. However, as these particular helplines were targeted towards mental wellbeing, the formation of positive interpersonal relationships may be more important for service-users using these services. For those contacting the breastfeeding helpline(s), it would appear that the provision of helpful, targeted support that encouraged them to continue breastfeeding, and that resolved and provided reassurance for their concerns were far more important than volunteers’ capacity for interpersonal connections.

This study identified that callers who were more determined to continue breastfeeding following the call were more likely to express overall satisfaction. However, the effect sizes suggest a weak association between these variables. Indeed, from the descriptive data only approximately 20% of callers felt that the helpline had made a difference in terms of whether they continued breastfeeding. Therefore, whilst ongoing telephone-based peer support appears to impact upon breastfeeding rates [19], the findings from this study suggest that one-off calls to the helpline were important in terms of encouraging, and making callers more determined, to breastfeed, rather than operating as a decisive factor for callers ongoing infant feeding decisions.

Whilst helpline evaluations are rare, the results of this study concur with other published studies in terms of high satisfaction rates, and satisfaction associated with the quality of information provided and efficacy of support on resolving specific issues [11-14]. Furthermore, similar to the study undertaken by Urbis Keys Young [12] the accessibility of the helpline, callers being provided with sufficient time and helpfulness of information provision were key features of satisfaction. To date, several studies have evaluated helpline support for breastfeeding women and reported on the reasons why women use a helpline service. As with our study, most calls related to very young infants (under two months old), and reasons for call were very similar to those reported here, such as concerns about milk supply, sore nipples, infant reflux and social pressures [17,22]. In one US-based study, however, the most common reason for calling related to the acquisition of and use of a breast pump, reflecting women’s economic situation in which an absence of maternity leave requires an early return to paid work [21]. Furthermore, whilst previous studies of breastfeeding helpline(s) have not identified characteristics that promoted user satisfaction, the results from the current study correspond with the evaluation of the Drugs in Breastmilk Helpline in that ‘reassurance’ was a key factor in the perceived helpfulness of the service [20].

The value women placed on accessibility of the helpline at the time they most needed it parallels the findings of Hoddinott and colleagues [24], who explored the infant experiences of women and their significant others from pregnancy until six months after birth. They noted that there were ‘pivotal points’ during the course of breastfeeding at which women made decisions related to infant feeding. The wellbeing of the family was central to this decision making. Our study shows that breastfeeding helplines can provide rapid input at a pivotal point when women may be vulnerable to introducing infant formula or discontinuing breastfeeding. Furthermore, the results from this study strongly resonate with the findings of a recent meta-synthesis of women’s perceptions and experiences of breastfeeding support [25] in terms of the importance of responsiveness, affirmation, encouragement and provision of realistic, practical support that is tailored to individual need. The meta-synthesis also identified the importance for women of the supporter having enough time and not appearing to be rushed [25]. Again, this was strongly identified in this helpline study.

### Caller demographic/call characteristics: association with overall satisfaction

The majority of caller demographics and call characteristics were not associated with overall satisfaction rates. To a large extent, this suggests that an equitable service is provided to callers of varying ages, parity, ethnic backgrounds and infant feeding status. Furthermore, the fact that satisfaction rates did not vary depending on which helpline was called, or which organisation received the call (BfN or ABM) provides reassurance that all callers receive a similar service, irrespective of whom they contact.

The finding that callers who were experienced at breastfeeding expressed higher levels of overall satisfaction than those who had no previous breastfeeding experience may be related to these callers contacting the helpline about less problematic issues and/or their issues may have been easier to resolve over the telephone. Moreover, the fact that overall satisfaction rates were lower for first-time breastfeeding mothers could potentially be attributable to their expectations around breastfeeding and/or experiencing more complex or multiple difficulties in establishing breastfeeding. Previous experiences of breastfeeding was also associated with the attitudinal variables of help and support (model 2), and not with the impact of support on caller wellbeing (model 3). These findings therefore suggest that the utility of the information and support in resolving specific issues was more important for women who were experienced at breastfeeding than promoting callers’ emotional wellbeing.

Overall, the data collated during the evaluation suggests that a number of calls are not answered; or that callers have to call multiple times before gaining access to the helpline service. Given the importance of rapid responsiveness identified by the callers in this study, with those finding it easy to access being significantly more likely to report high satisfaction, a key recommendation from this study is to improve access to the service, both by ensuring that callers have their call answered at the first attempt and by extending the service to provide 24 hour cover (as provided by the Australian Breastfeeding Association helpline). The first recommendation can be addressed as an operational issue while the second would require more careful consideration of the ways in which the volunteers work. Many of the volunteers are mothers of young children and may find voluntary night-time work impractical or unappealing. A payment scheme could therefore be introduced for an ‘out of hours’ service to facilitate the supporters being prepared to be called during the night, with a rota system to limit the number of ‘night’ shifts provided.

## Conclusion

This study suggests that many callers access UK breastfeeding helpline(s), and are highly satisfied with most features of the helpline service. Whilst the support does not necessarily impact on women’s breastfeeding decisions, this helpline service provides rapid, targeted, realistic, practical, and responsive support as well as affirmation and encouragement. The key features associated with overall satisfaction relate to the time the volunteers provided, how helpful the information was, callers being provided with the support that they needed, and feeling reassured following the call. A key recommendation from the study is that the helpline support needs to be easily accessible at all times to ensure that callers and their families can access support when needed. This may require consideration of the operationalization of call response management and extension to a 24 hour service.

## Competing interests

There are no financial or non-financial competing interests (political, personal, religious, ideological, academic, intellectual, commercial or any other) to declare in relation to this manuscript.

## Authors’ contributions

GT was involved in the design, data collection, analysis, reporting of the data and was lead author on the manuscript. FD and NC were involved in data collection, as well as the drafting and critical review of the manuscript. CS was involved in designing and analysis of the survey, reporting and interpretation of the results and drafting and critical review of the manuscript. All authors read and approved the final manuscript.

## Authors’ information

GT is a Research Fellow in the Maternal and Infant Nutrition and Nurture Unit (MAINN) at UCLan and was the project lead in undertaking this evaluation of the breastfeeding helpline(s) (May 2011 to February 2012). NC is a Research Assistant working in the MAINN unit at UCLAN and was one of the interviewers employed on this project. FD is Professor of Maternal and Infant Health and Director of MAINN. CS is a Senior Lecturer in Medical Statistics.

## Pre-publication history

The pre-publication history for this paper can be accessed here:

http://www.biomedcentral.com/1471-2393/12/150/prepub

## Supplementary Material

Additional file 1 **Table S1.** Regression models: Model 1 - Caller demographics/call characteristics; Model 2 - Model 1 plus attitudes and effectiveness of service characteristics; Model 3 - Model 1 plus caller wellbeing factors. Click here for file

## References

[B1] McEwen A Billings L Past use of and current satisfaction with a nurse-led hospital cardiac helpline Br J Card Nurs 2009 4 8 372 377

[B2] Shandley K Moore S Evaluation of Gambler’s Helpline: A Consumer Perspective Int Gambl Stud 2008 8 3 315 330 10.1080/14459790802409279

[B3] Reese RJ Conoley CW Brossartb DF Effectiveness of telephone counseling: A field-based investigation J Couns Psychol 2002 49 2 233 242

[B4] Coman GJ Burrows GD Evans BJ Telephone counselling in Australia: Applications and considerations for use Br J Guid Couns 2001 29 2 247 258

[B5] Dennis CL Kingston D A systematic review of telephone support for women during pregnancy and the early postpartum period J Obstet Gynecol Neonatal Nurs 2008 37 3 301 314 10.1111/j.1552-6909.2008.00235.x 18507601

[B6] Bryant RA An analysis of calls to a Vietnam veterans’ telephone counselling service J Trauma Stress 1998 11 3 589 596 10.1023/A:1024417031977 9690196

[B7] Takabayashi T Osada S Hiraguti S Onaka K Katakura N Ishigaki K Study on the effects of telephone counseling for family caregivers of demented patients Nippon Koshu Eisei Zasshi 2002 49 12 1250 1258 12607989

[B8] Gilbert H Sutton S Sutherland G Who Calls QUIT®? The characteristics of smokers seeking advice via a telephone helpline compared with smokers attending a clinic and those in the general population Public Health 2005 119 10 933 939 10.1016/j.puhe.2005.03.005 16083927

[B9] Hugo P Segwick P Black A Lacey H Telephone counselling - The EDA approach Eur Eat Disord Rev 1999 7 4 300 309 10.1002/(SICI)1099-0968(199908)7:4<300::AID-ERV279>3.0.CO;2-P

[B10] Dean A Scanlon K Telephone helpline to support people with breast cancer Nurs Times 2007 103 42 30

[B11] Boddy J Smith M Simon A Evaluation of Parentline Plus 2007 London: Home Office

[B12] Young UK National review of telephone counselling and web counselling services 2003 Canberra: Australian Government Department of Health and Ageing

[B13] Lim JM Sullivan E Kennedy D Mother safe: Review of three years of counselling by an australian teratology information service Aust N Z J Obstet Gynaecol 2009 49 2 168 172 10.1111/j.1479-828X.2009.00976.x 19432605

[B14] Dennis CL Hodnett E Kenton L Weston J Zupancic J Stewart DE Kiss A Effect of peer support on prevention of postnatal depression among high risk women: Multisite randomised controlled trial BMJ (Online) 2009 338 7689 280 283 10.1136/bmj.a3064 PMC2628301 19147637

[B15] Dennis CL Postpartum depression peer support: Maternal perceptions from a randomized controlled trial Int J Nurs Stud 2010 47 5 560 568 10.1016/j.ijnurstu.2009.10.015 19962699

[B16] Dale J Caramlau IO Lindenmeyer A Williams SM Peer support telephone calls for improving health Cochrane Database Syst Rev 2008 4 pub2 10.1002/14651858.CD006903 PMC7386897 18843736

[B17] Janssen PA Livingstone VH Chang B Klein MC Development and evaluation of a Chinese-language newborn needing hotline: A prospective cohort study BMC Pregnancy Childbirth 2009 9 10.1186/1471-2393-9-3 PMC2637834 19178746

[B18] Meglio GD McDermott MP Klein JD A randomized controlled trial of telephone peer support’s influence on breastfeeding duration in adolescent mothers Breastfeed Med 2010 5 1 41 47 10.1089/bfm.2009.0016 20043705

[B19] Dennis CL Hodnett E Gallop R Chalmers B The effect of peer support on breast-feeding duration among primiparous women: A randomized controlled trial CMAJ 2002 166 1 21 28 11800243 PMC99222

[B20] Rutter PM Jones W Enquiry analysis and user opinion of the Drugs inBreastmilk Helpline: A prospective study Int Breastfeed J 2012 6 10.1186/1746-4358-7-6 PMC3453512 22551014

[B21] Chamberlain LB Merewood A Malone KL Cimo S Philipp BL Calls to an inner-city hospital breastfeeding telephone support line J Hum Lact 2005 21 1 53 58 10.1177/0890334404272512 15681637

[B22] Wang SF Chen CH Related factors in using a free breastfeeding hotline service in Taiwan J Clin Nurs 2008 17 7 949 956 10.1111/j.1365-2702.2007.02111.x 18321292

[B23] McColl E Jacoby A Thomas L Soutter J Bamford C Steen N Thomas R Harvey E Garratt A Bond J Design and use of questionnaires: A review of best practice applicable to surveys of health service staff and patients Health Technol Assess 2001 5 31 i-v+1-250 10.3310/hta5310 11809125

[B24] Hoddinott P Craig LCA Britten J McInnes RM A serial qualitative interview study of infant feeding experiences: Idealism meets realism BMJ Open 2012 2 2 10.1136/bmjopen-2011-000504 PMC3307036 22422915

[B25] Schmied V Beake S Sheehan A McCourt C Dykes F Women’s Perceptions and Experiences of Breastfeeding Support: A Metasynthesis Birth 2011 38 1 49 60 10.1111/j.1523-536X.2010.00446.x 21332775

