# Supplementary material for: UK Breastfeeding Helpline support: An investigation of influences upon satisfaction
Source: BMC Pregnancy Childbirth. 2012 Dec 13;12:150. doi: 10.1186/1471-2393-12-150 (PMC3534517; doi:10.1186/1471-2393-12-150)
Supplement: Additional file 1 — Table S1. Regression models: Model 1 - Caller demographics/call characteristics; Model 2 - Model 1 plus attitudes and effectiveness of service characteristics; Model 3 - Model 1 plus caller wellbeing factors. [file 1471-2393-12-150-S1.docx]

### Additional File1: Table S1

### Regression models: Model 1 - Caller demographics/call characteristics; Model 2 – Model 1 plus attitudes and effectiveness of service characteristics; Model 3 – Model 1 plus caller wellbeing factors.

| **Characteristic** | **Model 1 (AR^2^=4.0%)** | **Model 2 (AR^2^=64.7%)** | **Model 3 (AR^2^=** 54.3**%)** |
| --- | --- | --- | --- |
|  | **Adjusted mean difference**  **(95% confidence interval)** | **Adjusted mean difference**  **(95% confidence interval)** | **Adjusted mean difference**  **(95% confidence interval)** |
| Previous breastfeeding experiences  Breastfed – not breastfed previously | 0.14 (0.04 to 0.23) | 0.07 (0.01 to 0.13) | 0.06 (−0.02 to 0.13) |
| Easy/difficult to get through to helpline  Very easy/easy – Difficult/very difficult  Very easy/easy – Neither easy nor difficult | 0.25 (0.08 to 0.41)  0.54 (0.32 to 0.76) | 0.12 (0.01 to 0 22)  0.23 (0.09 to 0.38) | 0.14 (0.01 to 0.26)  0.30 (0.12 to 0.47) |
| Volunteer had enough time  Strongly agree/agree – Disagree/strongly disagree  Strongly agree/agree – Neither agree nor disagree |  | 1.06 (0.77 to 1.34)  −0.23 (−0.52 to 0.07) |  |
| Information was helpful  Strongly agree/agree – Disagree/strongly disagree  Strongly agree/agree – Neither agree nor disagree |  | 0.76 (0.54 to 0.98)  0.68 (0.47 to 0.90) |  |
| Volunteer gave me the support I needed  Strongly agree/agree – Disagree/strongly disagree  Strongly agree/agree – Neither agree nor disagree |  | 0.54 (0.33 to 0.74)  0.43 (0.22 to 0.63) |  |
| Volunteer made me feel OK to carry on breastfeeding  Strongly agree/agree – Disagree/strongly disagree  Strongly agree/agree – Neither agree nor disagree |  | 0.43 (0.09 to 0.76)  0.48 (0.15 to 0.82) |  |
| Support met my expectations  Strongly agree/agree – Disagree/strongly disagree  Strongly agree/agree – Neither agree nor disagree |  | 0.94 (0.76 to 1.13)  0.32 (0.15 to 0.50) |  |
| Support helped to resolve my issues  Strongly agree/agree – Disagree/strongly disagree  Strongly agree/agree – Neither agree nor disagree |  | 0.25 (0.16 to 0.35)  0.24 (0.14 to 0.34) |  |
| Caller felt less stressed following the call  Strongly agree/agree – Disagree/strongly disagree  Strongly agree/agree – Neither agree nor disagree |  |  | 0.59 (0.35 to 0.83)  0.14 (−0.06 to 0.35) |
| Caller felt more confident following the call  Strongly agree/agree – Disagree/strongly disagree  Strongly agree/agree – Neither agree nor disagree |  |  | 0.66 (0.43 to 0.89)  0.16 (0.00 to 0.32) |
| Caller felt reassured following the call  Strongly agree/agree – Disagree/strongly disagree  Strongly agree/agree – Neither agree nor disagree |  |  | 0.91 (0.67 to 1.16)  0.71 (0.48 to 0.94) |
| Caller felt more determined to continue breastfeeding  Strongly agree/agree – Disagree/strongly disagree  Strongly agree/agree – Neither agree nor disagree |  |  | 0.18 (0.06 to 0.30)  0.21 (0.09 to 0.33) |
